# Can mobile health apps with smartphones and tablets be the new frontier of cognitive rehabilitation in older individuals? A narrative review of a growing field

**DOI:** 10.1007/s10072-023-07045-8

**Published:** 2023-09-13

**Authors:** Maria Grazia Maggio, Antonina Luca, Rocco Salvatore Calabrò, Filippo Drago, Alessandra Nicoletti

**Affiliations:** 1 Department of Biomedical and Biotechnological Sciences, Biological Tower, School of Medicine, University of Catania, Via S. Sofia 97, 95123 Catania, Italy; 2 IRCCS Centro Neurolesi “Bonino Pulejo”, Messina, Italy; 3 Department “G.F. Ingrassia”, Section of Neurosciences, University of Catania, Catania, Italy

**Keywords:** Cognitive domains, E-rehabilitation, Neurological disease, Older adults, Smartphone APP

## Abstract

**Introduction:**

A recent interesting field of application of telemedicine/e-health involved smartphone apps. Although research on mHealth began in 2014, there are still few studies using these technologies in healthy elderly and in neurodegenerative disorders. Thus, the aim of the present review was to summarize current evidence on the usability and effectiveness of the use of mHealth in older adults and patients with neurodegenerative disorders.

**Methods:**

This review was conducted by searching for recent peer-reviewed articles published between June 1, 2010 and March 2023 using the following databases: Pubmed, Embase, Cochrane Database, and Web of Science. After duplicate removal, abstract and title screening, 25 articles were included in the full-text assessment.

**Results:**

Ten articles assessed the acceptance and usability, and 15 articles evaluated the efficacy of e-health in both older individuals and patients with neurodegenerative disorders. The majority of studies reported that mHealth training was well accepted by the users, and was able to stimulate cognitive abilities, such as processing speed, prospective and episodic memory, and executive functioning, making smartphones and tablets valuable tools to enhance cognitive performances. However, the studies are mainly case series, case–control, and in general small-scale studies and often without follow-up, and only a few RCTs have been published to date.

**Conclusions:**

Despite the great attention paid to mHealth in recent years, the evidence in the literature on their effectiveness is scarce and not comparable. Longitudinal RCTs are needed to evaluate the efficacy of mHealth cognitive rehabilitation in the elderly and in patients with neurodegenerative disorders.

## Introduction

In 2021, in Europe, the prevalence of people aged more than 65 years was around 20.8% [1], and the percentage is expected to increase reaching 28.1% by 2050 [2]. The constant aging of the population is determining an increasing incidence of age-related neurodegenerative disorders, characterized by a cognitive decline (i.e., Parkinson’s disease—PD and Alzheimer’s disease—AD). Thus, the need to adapt the healthcare system to the multiple emerging needs, to improve assistance and guarantee the continuity of care is evident [3].

Moreover, during the SARS-COV-2 pandemic, the possibility of using innovative technologies to provide healthcare services at home has been emphasized [4–6]. In particular, telemedicine allowed the continuity of care and territorial assistance, without the physical presence of the therapist/clinician and the overload of hospitalized healthcare facilities, also favoring the reduction of the costs of the National Health Service [7, 8]. A recent interesting field of application of telemedicine/e-health involved smartphone apps. Hence, recent evidence has highlighted that mHealth through smartphones and tablets can be a useful tool for implementing effective and economical healthcare interventions, especially in the field of telecognitive rehabilitation. In fact, the devices have multiple functionalities, such as sensors, internet access, geolocation data, notifications, and clinical apps [9]. Furthermore, smartphones/tablets can provide support comparable to dedicated medical devices, without the burden and embarrassment of assistive devices [10]. However, despite the high diffusion of these technologies, their use in clinical practice is still poor [11]. Although research on mHealth began in 2014, there are still few studies using these technologies in healthy elderly and in neurological populations [12–14].

Indeed, it has been shown that the use of some smartphone apps may improve patient’s health, due to the use of gamification, colorful esthetics, point systems, social competitions (e.g., leaderboard), avatars, game rewards, story missions, which involve the user and improve physical activity [15, 16].

Considering that the interest in using the app in cognitive assessment and rehabilitation is growing [17–20], the aim of the present review was to summarize current evidence on the usability and effectiveness of the use of mHealth in older adults and in patients with neurodegenerative disorders.

### Search strategy

This review was conducted by searching for recent peer-reviewed articles published between June 1, 2010 and March 2023 using the following databases: Pubmed, Embase, Cochrane Database, and Web of Science. The goal of the research strategy was to track progress in using mHealth for cognitive domains in older adults with and without neurodegenerative disease. To this end, the comprehensive search was conducted using the following terms: “Cognitive Rehabilitation” AND “Smartphone” OR “Mobile App”; AND/OR “older adults” and “neurodegenerative disease.”

Inclusion criteria were (i) study participants aged older than 60, (ii) mHealth approach applied to cognitive rehabilitation, (iii) English language, and (v) published in a peer-reviewed journal. We excluded articles describing theoretical models, methodological approaches, algorithms, basic technical descriptions, and validation of experimental devices that do not provide a clear translation into clinical practice. In addition, we excluded (i) animal studies, (ii) studies focusing only on other innovative approaches (such as exergaming, or serious games without smartphones or tablets), or (iii) on assessment or monitoring.

Titles and abstracts were screened independently. Relevant articles were then fully assessed. Disagreements over the article selection have been solved by discussion and with the supervision of a senior researcher.

The list of articles was then refined for relevance, revised, and summarized, with the key themes identified from the summary based on the inclusion/exclusion criteria. The following information was considered: authors, year and type of publication (e.g., clinical trials, pilot study), characteristics of the participants involved in the study, and purpose of the study.

## Results

**Fig. 1:**
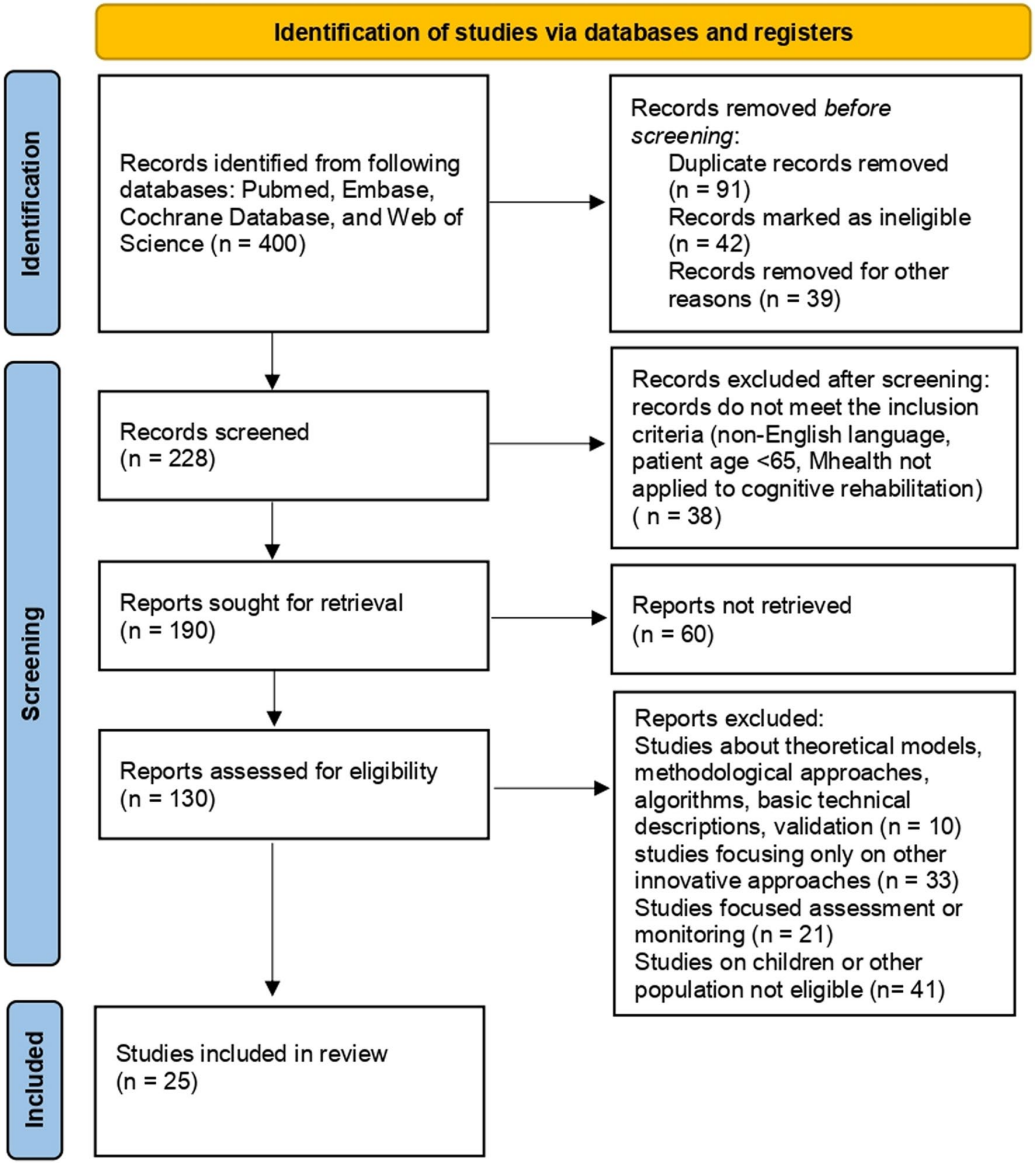
PRISMA 2020 flow diagram for new synthematic reviews which included searches of databases and registers only. From: Page MJ, Mckenzie JE, Bossuyt PM, Boutron I, Hoffman TC, Mulrow CD, et al. The PRISMA 2020 statement an updated guideline for reporting systematic reviews. BMJ 2021; 372:n71 https://doi.org/10.1136/bmj.n71

The database search produced a total of 400 titles. After duplicate removal and abstract and title screening, 25 articles were included in the full-text assessment. A flowchart of study selection is presented in Fig. 1. The main findings of the selected articles are reported in Table 1.

**Table 1:** shows the principal studies concerning telerehabilitation via mHealth

Type	Authors	Study design	Sample characteristics	Device and training	Major findings
*Acceptance and usability*	Vaportzis et al. (2017)	RCT	43 older adults	Tablet training weekly 2-h class for 10 weeks	Tablet training was well accepted and could boost processing speed
	Heinz et al. (2013)	Focus group	30 older adults	3 separate focus groups (an independent apartment complex, a rural community, and exercise program participants)	Older adults were willing to use new technologies if the utility of the tools was high and enabled them to overcome feelings of inadequacy
	Petrovčič et al. (2019)	Exploratory study	617 older adults	Cycle of Technology Acquirement by Independent-Living Seniors (C-TAILS) model phone interview	How the patient uses the smartphone on a daily living and the breadth of smartphone functionality influences the patient's ability to use assistive apps
	Chan et al. (2016)	Exploratory study	54 older adults	Extensive iPad training 15 h/week for 3 months	iPad apps could increase episodic memory and processing speed
	Vaportzis et al. (2017)	Focus group	18 older adults	Tablet training weekly 2-h class for 10 weeks	Learning using a tablet was considered “useful.” The major concern of patients was the lack of clarity in instructions and support
	Vaportzis et al. (2018)	Mixed methods study	43 older adults 14 healthy older adults	Tablet training weekly 2-h class for 10 weeks	Participants became more confident with tablet and enjoyed downloading applications
	MenéndezÁlvarez-Dardet et al. (2020)	Exploratory study	212 Spanish older adults	Active PC Not specified	Conflicting results. Possible age-related findings
	Benge et. al. (2020)	Exploratory study	53 older adults with cognitive impairment 44 care partners 204 control	Smartphone Daily use	Smartphone apps might be a feasible intervention for some patients
	Maggio et al. (2022)	Feasibility study	16 PD patients	Neuronation brain training 3 times a week for six weeks	The tool has good usability and feasibility
*Effectiveness in the elderly*	Jang et al. (2021)	RCT	389 non-demented elderly	Application-based Cognitive Training at Home (ACTH) for 12 months	Global cognition improvement in non-demented elderly individuals
	Vaportzis et al. (2017)	RCT	43 older adults	Tablet training weekly 2-h class for 10 weeks	Tablet training was well-accepted and could boost processing speed
	Chan et al. (2016)	Exploratory study	54 older adults	Extensive iPad training 15 h/week for 3 months	iPad apps could increase episodic memory and processing speed
	Heinz et al. (2013)	Focus group	30 older adults	3 separate focus groups (an independent apartment complex, a rural community, and exercise program participants)	Participation in engaging activities characterized by new learning promoted the improvement of various cognitive skills, such as memory and executive functioning
	Xavier et al. (2014)	Longitudinal	6442 older adults	Internet Not specified	Digital literacy could reduce cognitive decline among people aged 50–89
	Yuan et al. (2019)	Exploratory study	3230 older adults	Smartphone Not specified	Higher smartphone use was positively associated with increased cognitive functions. Gender differences were found in the effect of smartphone use on visuospatial skills and memory
	Tun et al. (2010)	Exploratory study	267 adults	Computer Not specified	Frequent computer use can promote good cognitive function, particularly for executive control, from adulthood to old age, especially for subjects with lower cognitive ability
	Silber et al. (2016)	Experimental study	27 older adults	Computer Not specified	Less daily computer use was associated with smaller brain volume in regions of memory
	Zilberman et al. (2016)	Pilot study	8 older adults	8-week educational tablet training program	Participants who were actively engaged in a structured group with individual support were more likely to transfer skills into their environments and daily routines to promote performance and occupational performance satisfaction
*Effectiveness in patients with neuro-degenerative disorders*	Scullin et al. (2022)	RCT	52 older adults with mild dementia	Digital voice recorder app or a reminder app 4-week randomized controlled trial	Seniors with cognitive impairments improved memory strategies through smartphone training, which enhanced prospective memory
	Kraepelien et al. (2020)	RCT	77 PD patients	Internet-based cognitive behavioral therapy 10 weeks	Smartphone training was useful as an adjunct to standard medical treatment and improved the cognitive functioning of individuals with PD
	Wu et al. (2019)	Experimental study	112 older elderly 127 MCI 84 AD	Computer and a touchscreen device Daily use	Participants who did not use technologies daily had reductions in global cognitive function, processing speed, short-term memory and executive function
	Aghanavesi et al. (2017)	Experimental study	19 PD patients 22 healthy controls	Smartphone Not specified	Using smartphone, it is possible to intervene effectively and evaluate the skills of PD subjects
	Nicosia et al. (2022)	Experimental study	268 normal older adults 22 individuals with mild dementia	Smartphone 4 times per day over 7 consecutive days	Smartphone training was reliable and valid and represents a feasible tool for boosting cognitive domains
	El Haj et al. (2021)	Experimental study	22 patients with mild AD	Smartphone-based calendars 3 weeks period	Smartphone applications may be useful for boosting prospective memory in AD
	Pang et al. (2021)	Experimental study	42 patients with AD	Smartphone-based calendar training and walking exercise 12 weeks	Smartphone-based training improved cognitive function and have the potential as non-pharmacological interventions to boost cognitive functioning in women suffering from subjective cognitive decline

*AD* Alzheimer disease, *RCT* randomized controlled trial, *PD* Parkinson disease

### Acceptance and usability

Ten articles were included: 8 articles enrolling healthy elderly [21–31] and 2 articles enrolling patients with neurodegenerative disorders (1 article assessing elderly with cognitive impairment [32], and 1 article assessing patients with Parkinson’s disease-PD [14]). Out of these, only the study performed b Vaportzis et al. [21], enrolling 43 seniors and reporting a good acceptance and usefulness of tablet training was an RCT. However, data on “familiarity” with smartphones and tablets remains controversial [21–32]. Heins et al. carried out a study on 30 elderly patients, reporting that the use of technology was “viewed positively” since it helped maintain independence and quality of life [22]. Moreover, older individuals without cognitive decline showed interest in using their smartphones/tablets as a cognitive aid (e.g., reminders, alarm clocks, calendars). In addition, patients with cognitive impairment were found to have adequate acceptance and usability of devices to facilitate cognitive functioning [14, 31]. On the contrary, other studies have pointed out that younger patients had better outcomes through the use of mHealth devices than older ones [25, 26], probably due to a lack of confidence in electronic devices. The characteristics of some devices, such as internet signal problems and poorly understood interface, can create difficulties of use in the elderly, especially with cognitive deterioration. Indeed, in studies performing a training section before proposing the use of devices for cognitive support-rehabilitation, a good acceptance and usability of smartphones, and even more tablets, were reported [24, 27–31]. Bier et al. found that subjects with and without cognitive impairment had generalized the skills learned during training interventions to other smartphone and tablet functions, using other apps in daily life [31]. Confirming this data, Imbeault et al. reported that cognitively impaired subjects, in addition to the cognitive stimulation app, installed other apps such as diaries, or recipe apps to improve self-esteem [25]. Furthermore, we previously reported good feasibility and usability of a 6-week cognitive rehabilitation protocol based on the non-immersive virtual reality telecognitive app in non-demented PD patients [14].

### Effectiveness of telerehabilitation via mHealth in the elderly

Eight articles were included, of which 2 RCT studies: Vaportzis et al. [21] reported that table training improved processing speed; Jang et al. enrolled 389 non-demented elderly volunteers and reported that home cognitive training via smartphone improved cognitive performances in terms of global cognitive functioning, language, and memory [33]. In a study enrolling 30 elderly individuals, Heintz et al. reported that participation in engaging activities characterized by new learning promoted the improvement of various cognitive skills, such as memory and executive functioning [22]. Xavier et al. in a longitudinal study enrolling more than 6400 elderly individuals demonstrated that increased Internet/email use was associated with significant improvement in memory performances [34]. Other studies have shown that mHealth enables improvements in executive functioning, such as processing speed and mental flexibility, in individuals with and without dementia. In particular, Chan et al. [27] conducted a study on 18 elderly individuals with no computer knowledge, reporting that, after training on tablet use, an improvement in episodic memory and processing speed was observed. Confirming these data, Yuan et al. noted that older adults without cognitive impairment through smartphone use showed more significant improvements in all cognitive domains, especially executive functions, than non-smartphone users [35]. Moreover, Tun and Lachman in a study of 2671 adults demonstrated that computer use can stimulate executive functions, especially the ability to switch attention and alternate attention [36]. Similarly, Kesse-Guyot et al. in a longitudinal cohort study found that older people using mHealth devices showed increases in episodic memory and executive function [37]. Furthermore, it has been observed that the knowledge acquired through mHealth training can be maintained for a long time, both in subjects with and without cognitive impairment [38–42]. Interestingly, the Intelligent Systems for Assessing Aging Change study on longitudinal aging has shown that the reduced use of technologies (PCs, tablets, smartphones) was associated with a smaller hippocampal volume and worse performance on memory and functioning executive in older adults with cognitive impairment [38].

### Effectiveness of telerehabilitation via mHealth in patients with neurodegenerative disorders

Seven articles [43–50], including 2 RCT studies [43, 44], were included. Scullin et al. sin a study on 52 elderly people with mild dementia reported that smartphone training could stimulate memory, especially prospective memory, by learning new strategies [43]. Kraepelien et al. enrolled 77 PD patients and pointed out that smartphone training was helpful as an adjunct to standard medical treatment to improve cognitive functioning [44]. Indeed, it has been shown that mHealth training could have better cognitive outcomes from using smartphones and tablets [43–50]. Wu et al. in a study of elderly people with MCI observed that the control group, who had not used technologies, presented a reduction of global cognitive functioning, processing speed, short-term memory, and executive function [45]. Indeed, Aghanavesi et al. in a study on PD patients demonstrated that through the use of smartphones, it was possible to effectively intervene in cognitive skills [46]. These findings were also supported by Nicosia et al. [47]. The authors conducted a study on 268 cognitively normal seniors (aged 65–97 years) and 22 individuals with mild dementia showing that smartphones have the potential to intervene in the first phases of AD improving short-term memory, processing speed, and working memory [47]. These results were confirmed by El Haj et al. who highlighted the positive effect of using smartphone-based calendars on prospective memory in AD [49]. Similar findings were reported by Pang and Kim, performing a study on smartphone-based calendar training and walking exercise regimen in 42 postmenopausal women with subjective cognitive decline [50].

## Discussion

Several studies supported the feasibility and efficacy of mHealth in both older individuals [21–31, 33–42] and patients with neurodegenerative disorders [32, 43–49]. Moreover, studies reported that the use of mHealth training was able to stimulate cognitive abilities, such as processing speed, prospective and episodic memory, and executive functioning [21, 43–49] making smartphones and tablets valuable tools to enhance cognitive performances.

Some authors have shown that the use of mHealth could improve cognitive abilities and allow the generalization of outcomes in daily life, even in the presence of neurodegenerative disorders [19, 20, 31].

Unfortunately, it should be noted that, although the growing interest in this topic, literature data on the use of mHealth in older adults with or without neurodegenerative disorders is still scarce. It should be noted that the majority of selected studies were case–control carried out on small sample and that methodological differences such as the study population (healthy elderly versus cognitively impaired), and type of apps do not allow the comparison across the studies. Furthermore, few RCTs have been performed—in fact, to the best of our knowledge, only 2 RCTs on the effectiveness of smartphone training on older adults, and 2 RCTs related to the efficacy of smartphone tools in neurodegenerative disorders are available [28, 43–45]. However, literature data suggest that it would be useful to carry out specific training to increase the use of technologies, favor the effects of cognitive rehabilitation in older individuals with and without cognitive decline [28, 43–45], improve “familiarity” with technological tools [48], reducing anxiety about technology or technophobia [25].

In conclusion, the present review underlines that despite the great attention paid to mHealth in recent years, especially after the COVID-19 pandemic. Longitudinal RCTs are needed to evaluate the efficacy of mHealth cognitive rehabilitation in healthy elderly and in patients with neurodegenerative disorders.

## Data Availability

Data will be available on request to the corresponding author.
